# Single-cell long noncoding RNA (lncRNA) transcriptome implicates MALAT1 in triple-negative breast cancer (TNBC) resistance to neoadjuvant chemotherapy

**DOI:** 10.1038/s41420-020-00383-y

**Published:** 2021-01-25

**Authors:** Hibah Shaath, Radhakrishnan Vishnubalaji, Ramesh Elango, Shahryar Khattak, Nehad M. Alajez

**Affiliations:** 1 College of Health & Life Sciences, Hamad Bin Khalifa University (HBKU), Qatar Foundation (QF), Doha, Qatar; 2 Cancer Research Center, Qatar Biomedical Research Institute (QBRI), Hamad Bin Khalifa University (HBKU), Qatar Foundation (QF), PO Box, 34110 Doha, Qatar; 3 Stem Cell Core, Qatar Biomedical Research Institute (QBRI), Hamad Bin Khalifa University (HBKU), Qatar Foundation (QF), PO Box, 34110 Doha, Qatar

**Keywords:** Breast cancer, Long non-coding RNAs, Breast cancer

## Abstract

Cumulative evidence suggests added benefit for neoadjuvant chemotherapy (NAC) in a subset of triple-negative breast cancer (TNBC) patients. Herein we identified the long noncoding RNA (lncRNA) transcriptional landscape associated with TNBC resistance to NAC, employing 1758 single cells from three extinction and three persistence TNBC patients. Using Iterative Clustering and Guide-gene Selection (ICGS) and uniform manifold approximation and projection (UMAP) dimensionality reduction analysis, we observed single cells derived from each patient to largely cluster together. Comparing the lncRNA transcriptome from single cells through the course of NAC treatment revealed minimal overlap based on lncRNA transcriptome, suggesting substantial effects of NAC on lncRNA transcription. The differential analysis revealed upregulation of 202 and downregulation of 19 lncRNAs in the persistence group, including upregulation of five different transcripts encoding for the MALAT1 lncRNA. CRISPR/Cas9-mediated MALAT1 promoter deletion in BT-549 TNBC model enhanced sensitivity to paclitaxel and doxorubicin, suggesting a role for MALAT1 in conferring resistance. Mechanistically, whole transcriptome analysis of MALAT1-KO cells revealed multiple affected mechanistic networks as well as oxidative phosphorylation canonical and angiogenesis functional category. Interestingly, lncRNA profiling of MALAT1-depleted TNBC also revealed a number of altered lncRNAs in response to MALAT1 deletion, suggesting a reciprocal relationship between MALAT1 and a number of lncRNAs, including NEAT1, USP3-AS1, and LINC-PINT, in TNBC. Elevated expression of MALAT1, USP3-AS1, and LINC-PINT correlated with worse clinical outcomes in BC patients. Our data revealed the lncRNA transactional portrait and highlighted a complex regulatory network orchestrated by MALAT1 in the context of TNBC resistance to NAC therapy.

## Introduction

Breast cancer (BC) is the most prevalent type of cancer and the most common cause of cancer-related deaths among women worldwide^1^. Despite many successes in the field of BC therapy, treatment regimen and the response rate among various molecular subtypes varies. Triple-negative breast cancer (TNBC) is characterized by the absence of receptors commonly used for classification and therefore as targets. These include the estrogen receptor (ER), human epidermal growth factor receptor-2 (HER2), and progesterone receptor (PR).

Neoadjuvant chemotherapy (NAC) remains the gold standard form of therapy for TNBC patients, with limited effectiveness, narrower durations of response, and considerably toxic profiles^2^. As a highly heterogeneous BC subtype, there are very few options for treating TNBC patients that confer resistance to conventional chemotherapy^3^. Not only does TNBC lack targeted therapy options, but it is also the most aggressive subtype with higher incidences of metastasis, early recurrence and poor overall survival, accounting for around 15–20% of all BC cases^4–6^. Unfortunately, only around one in three patients successfully respond to the treatment^7^, making it crucial to find alternative therapies by exploring alternative avenues regarding TNBC resistance mechanisms, which remains largely unknown.

Despite the overall chemo-sensitivity of TNBC to NAC compared to non-TNBC, early complete response (CR) in TNBC patients does not result in higher overall survival due to the high risk of metastatic relapse during the first 5 years^8^. The development of resistance in TNBC can range from a staggering 40–80% in the first three years following treatment^6,9^, which makes being able to predict the response of each patient a valuable tool clinically, financially, and time wise, in efforts to bring patients better personalized medical care. To gain insight into the mechanisms of resistance in TNBC, our understanding of whether it comes from the expansion and adaptation of cells already in existence in the cancer cell population (adaptive resistance) or rather from cells arising as a result of new mutations (acquired resistance), has to be established.

Long non-coding RNAs (lncRNAs) have been identified in recent years to be important players in multiple biological functions, shaping our understanding of processes such as embryonic development and gene expression regulation^10^. Differential expression of some lncRNAs has been associated with several types of cancer^11^. In recent studies, many lncRNAs including SOX21-AS1, HOST2, HUMT, XIST, FAM83H-AS1, and LINC00173 have been identified in roles associated with the onset and progression of TNBC through different pathways and processes^9,12–15^. To draw a bigger picture and identify those associated with NAC resistance, RNAseq data of single cells derived from patients who exhibited a large degree of resistance to NAC prior and post NAC treatment were analyzed using a range of bioinformatics tools. The use of single-cell analysis is particularly beneficial in the breakdown of cell population heterogeneity rather than identifying an average response that is not representative of any one cell type. In this scenario, we can identify cell populations within the heterogenic TNBC population contributing to NAC resistance, and to target those unique signatures for therapy.

The main aim of this study was to identify the transcriptional landscape of TNBC resistance to NAC treatment at the single-cell level, utilizing lncRNA-based molecular classification. Over 200 transcripts were found to be differentially expressed in the persistence patients compared to the extinction group, where several of those transcripts were found to encode for MALAT1 lncRNA. We subsequently used CRISPR/CAS9-mediated genome editing to delete the promoter of MALAT1 gene in TNBC cell models, which gave rise to a number of affected mechanistic networks with alterations in other lncRNA expression patterns, confirming MALAT1s crucial role in various cellular processes and its potential association with NAC resistance in TNBC.

## Results

### Iterative Clustering and Guide-gene Selection (ICGS) of TNBC-derived single cells prior to neoadjuvant therapy

**Fig. 1:**
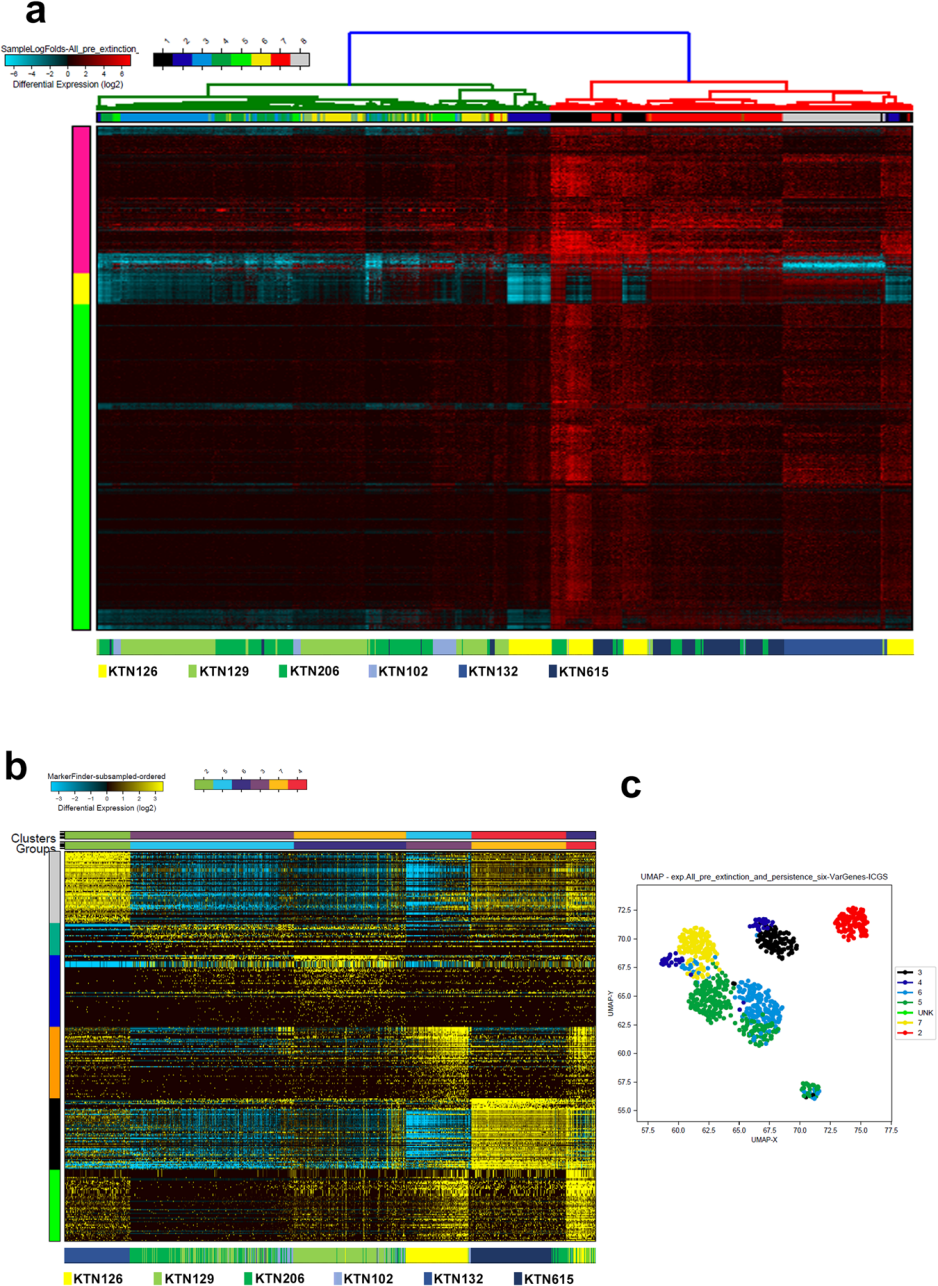
Iterative Clustering and Guide-gene Selection (ICGS) of TNBC-derived single cells pre neoadjuvant therapy. **a** Hierarchical clustering of 872 single cells derived from three extinction (KTN126, KTN129, and KTN206) and three persistence (KTN102, KTN132, and KTN615) triple-negative breast cancer (TNBC) prior to neoadjuvant chemotherapy based on lncRNA transcriptome. **b** Unsupervised single-cell population identification using ICGS algorithm conducted on 577 single cells derived from the extinction (KTN126, KTN129, and KTN206) and 295 cells derived from the persistence (KTN102, KTN132, and KTN615) group prior to neoadjuvant treatment. Data are presented as heat map with the corresponding single-cell cluster on top. Color scale displays differential gene expression (log2). The lower legend indicated the source of each cell. **c** Uniform manifold approximation and projection (UMAP) dimensionality reduction analysis of 872 single cells from the same cohort prior to neoadjuvant treatment.

In order to identify potential lncRNA-based signatures predictive of response to NAC treatment, sequencing data derived from 872 single cells from three extinction and three persistent TNBC patients prior to neoadjuvant therapy were subjected to hierarchical clustering. Clustering patterns displayed that single cells derived from the extinction and the persistence group clustered into eight different subgroups (*x*-axis), which clustered into three different clusters (*y*-axis) based on their lncRNA profile. The lncRNA profile associated with each subgroup is shown in Supplementary Table 1 and Fig. 1a. We observed single cells derived from each patient to largely cluster together. Patients that exhibited extinction phenotype, in general, clustered together while patients exhibiting persistence phenotype clustered separately (Fig. 1a). Interestingly, we also observed some cells from the persistence group to cluster with the extinction group and vice versa, suggesting a certain degree of overlap in single cells derived from the two patient groups based on their lncRNA transcriptome. Transcriptome data from the same patient cohort were subjected to the ICGS hierarchical clustering algorithm, which allows us to predict cell states based on their common transcriptomic profile (Fig. 1b). ICGS analysis further revealed heterogeneity in the lncRNA transcriptome of the extinction and persistence groups, where cells from each patient group tend to cluster together with enrichment of appropriate functional oriented clear population on the *y*-axis (6 rows) and the corresponding single-cell cluster on the top *x*-axis (6 columns) (Fig. 1b). The lncRNA transcriptome associated with the indicated clustering group is provided in supplementary table 2. This cellular heterogeneity is alternatively represented by the uniform manifold approximation and projection (UMAP) in Fig. 1C, which confirms the clustering of each cohort with its respective phenotype group (extinction vs persistence). UMAP analysis revealed 7 sub clonal populations including one unknown phenotype in the heterogeneity.

### ICGS analysis of TNBC-derived single cells pre and mid neoadjuvant therapy

**Fig. 2:**
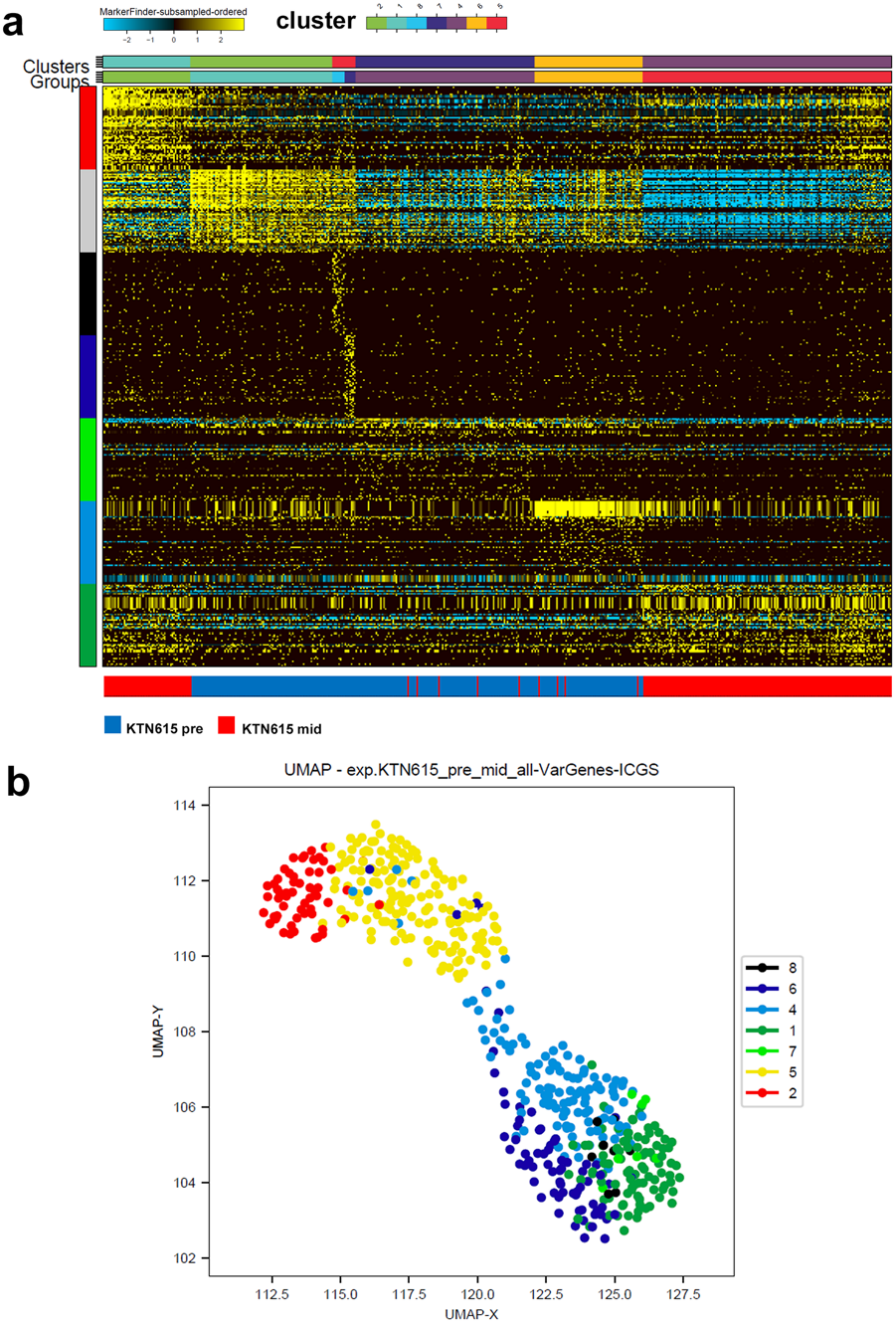
Iterative Clustering and Guide-gene Selection (ICGS) of TNBC-derived single cells pre and mid neoadjuvant therapy. **a** Unsupervised single-cell population identification using ICGS algorithm conducted on 284 single cells derived from KTN615 pre (blue color legend) and 227 single cells derived from the same patient (KTN615) mid (red color legend) neoadjuvant treatment who exhibited persistence phenotype. **b** Uniform manifold approximation and projection (UMAP) dimensionality reduction analysis of 284 single cells derived from KTN615 pre (blue color legend) and 227 single cells derived from the same patient (KTN615) mid (red color legend) neoadjuvant treatment who exhibited persistence phenotype.

Delving deeper into the transcriptional landscape of patients that show persistence both before and after neoadjuvant therapy highlighted important changes to aberrant lncRNA expression in these patients. Patient KTN615 was chosen for this comparison as they displayed a larger degree of resistance compared to the other two patients based on data by Kim et al.^16^. Data from 284 (pre-treatment) and 227 (mid-treatment) single cells taken from KTN615 were used to conduct unsupervised Single-Cell Population Identification using the ICGS algorithm. Interestingly, clustering analysis heat map (Fig. 2a), revealed clear segregation of the pre and mid NAC cells, suggesting a complete shift of the lncRNA transcriptional profile post NAC treatment. There were a few exceptions where several cells from the mid NAC clustered within the pre NAC cells, suggesting a possible persistence of single cells during NAC treatment in this patient. Cells from KTN615 pre and mid NAC cohort tend to cluster together with enrichment of seven cell clusters according to the functional categories on the y-axis and the corresponding single-cell cluster on top *x*-axis (7 columns). The list of lncRNAs correlating with the indicated clusters pre and mid NAC treatment is provided in supplementary table 3. Cellular heterogeneity was further displayed with seven different populations using the UMAP analysis (Fig. 2b).

### Comparative analysis of the lncRNA transcriptional landscape in persistence and extinction TNBC-derived single cells pre-neoadjuvant treatment

**Fig. 3:**
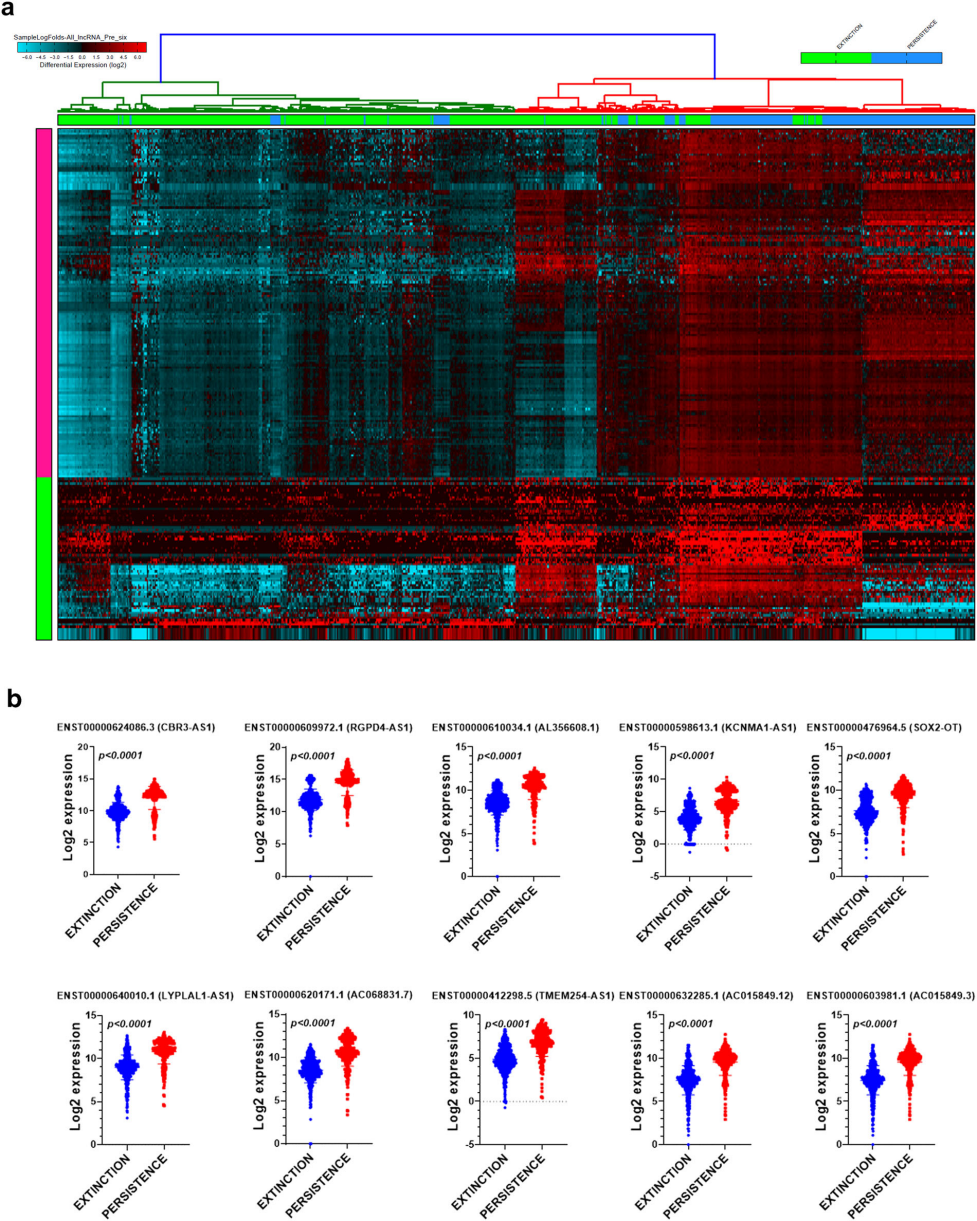
Comparative analysis of the transcriptional landscape in persistence and extinction TNBC-derived single cells pre-neoadjuvant treatment. **a** Hierarchical clustering of TNBC-derived single cells from persistence (*n* = 295) and extinction (*n* = 577) group prior to neoadjuvant treatment. Each column represents one cell and each row represents lncRNA transcript. The expression level of each transcript (log2) in a single cell is depicted according to the color scale. **b** Validation of selected number of upregulated lncRNA transcripts in a second cohort of 580 and 297 single cells derived from extinction and persistence TNBC patients, respectively. Data are presented as scatter plots with *p* values indicated on each plot (two-tailed *t*-test).

To gain more insight into the lncRNA profile which could discriminate the persistence from the extinction phenotype in response to NAC treatment, the lncRNA expression profile of single cells derived from three persistence (295 cells) and three extinction (577 cells) patient samples, were compared (Fig. 3a). Hierarchical clustering shows two clear cell clusters (pink and green), each column represents a single cell and each row represents a lncRNA expression (log2) that is represented according to the color scale, which revealed the up- and downregulation of several lncRNAs in the persistence group (supplementary table 4). To validate the findings from this discovery cohort, the expression of top altered lncRNAs was validated in a second cohort of 580 cells (extinction cohort) and 297 cells (persistence cohort) from the same TNBC patient. The expression of selected upregulated lncRNA transcripts (CBR3-AS1, RGPD4-AS1, AL356608.1, KCNMA1-AS1, SOX2-OT, LYPLAL1-AS1, AC068831.7, TMEM254-AS1, AC015849.12, and AC05849.3) in the persistence and extinction validation cohort are shown in Fig. 3b.

### Effect of CRISPR/Cas9 mediated-MALAT1 promoter deletion on BT-549 colony-forming and paclitaxel/Doxorubicin sensitivity

**Fig. 4:**
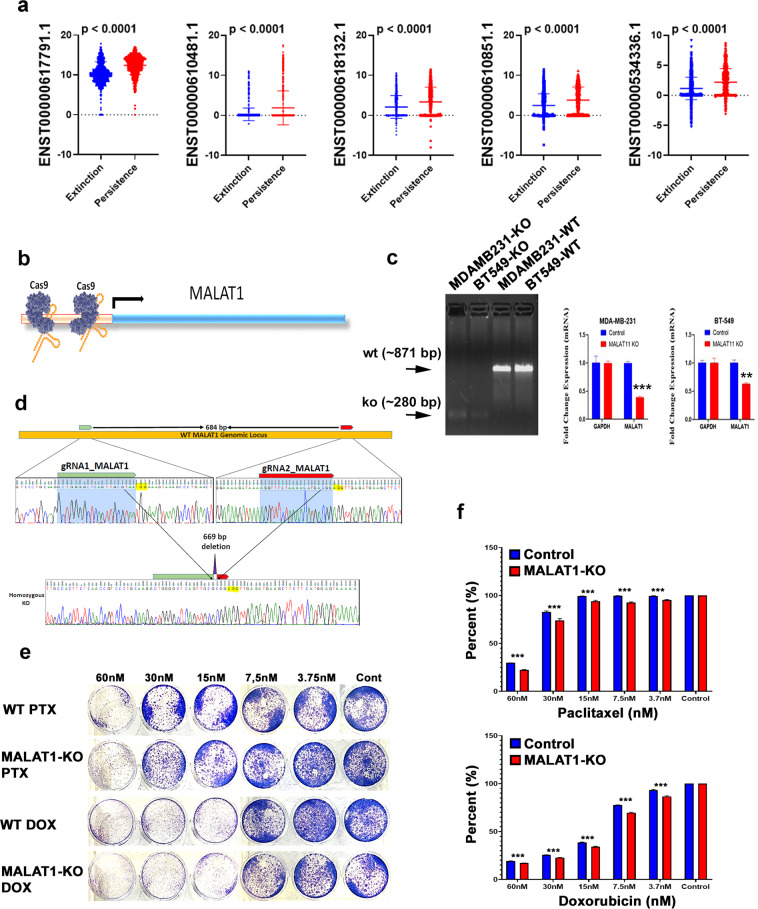
Effect of Cas9 mediated-MALAT1 knockdown on BT-549 colony-forming and paclitaxel/ Doxorubicin sensitivity. **a** Expression of the indicated MALAT1 transcripts in 1157 single cells derived from the extinction (KTN126, KTN129, and KTN206) and 592 single cells derived from the persistence (KTN102, KTN132, and KTN615) TNBC patients. Data are presented as a scatter plot with *p* values indicated on each plot (two-tailed *t*-test). **b** Strategy to knockdown MALAT1 promoter using dual guide RNA and Cas9 protein. **c** Successful deletion of the MALAT1 promoter using CRISPR/Cas9 in MDA-MB-231 and BT-549 TNBC model. QRT-PCR for MALAT1 expression in BT-549 wt and MALAT1-KO cell models. Data are presented as mean ± SD, *n* = 3. **p < 0.005, ***p < 0.0005. **d** Sanger sequencing confirming intended homozygous deletion of MALAT1 promoter in MDA-MB-231 cells. **e** Clonogenic assay for BT-549 and BT-549-MALAT1-KO in the presence of different concentration of Paclitaxel or Doxorubicin. **f** Quantification of CFU data from (**e**). Data are presented as mean ± SD, *n* = 4. ***p < 0.0005.

Interestingly, we observed several lncRNA transcripts (ENST00000617791.1, ENST00000610481.1, ENST00000618132.1, ENST00000610851.1, and ENST00000534336.1) all derived from the MALAT1 locus, were upregulated in the persistence group (Fig. 4a), suggesting a possible role for MALAT1 in driving TNBC NAC resistance. Given the differential upregulated expression of MALAT1 in the persistence phenotype, we subsequently sought to elucidate the role for MALAT1 in mediating TNBC chemo-resistance through the implementation of CRISPR/CAS9 genome editing technology to delete the promoter of MALAT1 gene in the BT-549 TNBC model. To achieve this, two guide RNAs targeting the large MALAT1 promoter were synthesized as described in the material and methods section and illustrated in Fig. 4b. PCR of the MALAT1 promoter revealed a complete deletion of the intended genomic region in the BT-549 and MDA-MB-231 MALAT1-KO model compared to the parental cells (Fig. 4c). An intense band at around 871 base pairs in the wild type represents the unaffected MALAT1 promoter, compared to the ~280 base pairs region identified in the MALAT-KO models, demonstrating complete deletion of the intended genomic region (Fig. 4c). Concordantly, qRT-PCR confirmed remarkable downregulation of MALAT1 expression levels in the MALAT1-KO models compared to the WT, using GAPDH as a loading control (Fig. 4c). MALAT1 promoter deletion was further verified using Sanger sequencing (Fig. 4d). Colony-forming unit (CFU) assay was utilized to look at the numbers of viable cells with the ability to undergo binary fission to form distinct colonies in the presence of different concentrations of Paclitaxel in both the MALAT1 knockout and parental BT-549 cell models. Figure 4e shows the BT-549 MALAT1-KO and parental models and their colony formation in response to a serial dilution of Paclitaxel or doxorubicin; 60, 30, 15, 7.5, 3.7 nM and no drug (control). At a concentration of 30 nM and lower, the MALAT1-KO BT-549 cells formed fewer colonies compared to the BT-549 wt model (Fig. 4e), suggesting MALAT1 depletion to enhance the efficacy of NAC. Quantification of CFU data is presented in Fig. 4f.

### Transcriptome analysis of MALAT1-KO TNBC cells revealed global changes in mRNA and lncRNA transcriptome

**Fig. 5:**
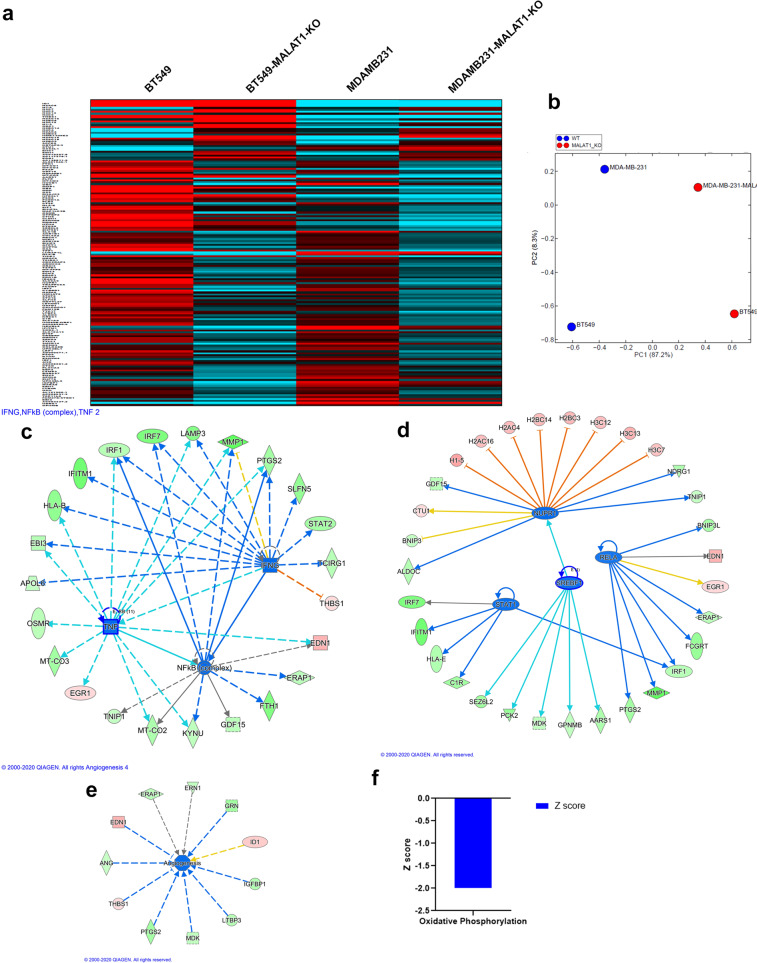
Alteration of gene expression in MALAT1-KO TNBC cells. **a** Heatmap depicting the expression of upregulated (32) and downregulated (129) genes (1.5 ≤ fc ≥ 1.5) in the BT-549 and MDA-MB-231 TNBC models using whole transcriptome analysis. **b** Principal component analysis (PCA) illustrating the segregation of BT-549, BT-549-MALAT1-KO, MDA-MB-231, and MDA-MB-231-MALAT1-KO based on PC1 and PC2. Ingenuity pathway analysis (IPA) on the 161 common differentially expressed transcripts in MALAT1-KO TNBC models highlighting suppression of INFG, NFKB, and TNF networks (**c**). Suppression of NUPR1, STAT1, RELA, and SREBF1 transcription regulator (**d**), angiogenesis functional category (**e**), as well as oxidative phosphorylation canonical pathway (**f**) in MALAT1-KO TNBC cells.

Whole transcriptome analysis comparing the parental cell and MALAT1-KO cell populations for BT-549 and MDA-MB-231 were performed to gain a more comprehensive insight into gene expression changes associated with MALAT1 knockdown. Figure 5a shows a heat map of the commonly altered mRNAs in the context of MALAT1 knockdown in both BT-549 and MDA-MB-231 TNBC models. Thirty-two genes were upregulated (represented by red bars in the heat map) whereas 129 genes were downregulated (blue) in our MALAT-KO models in comparison to their parental models (Supplementary Table 5). PCA analysis confirmed the segregation of the MALAT1-KO from wt cells based on PC1 and PC2, with about 87% of the variation attributed to PC1 (Fig. 5b).

The 161 differentially expressed transcripts in the MALAT1-KO models were subjected to Ingenuity Pathway Analysis (IPA). This, in turn, highlighted several pathways whereby key upstream networks such as INFG, NFKB, and TNF were suppressed in MALAT1-KO TNBC models (Fig. 5c). The NUPR1, STAT1, RELA, and SREBF1 transcription networks were predicted to be suppressed in the MALAT1-KO models, with the affected gene partners within each network indicated (Fig. 5d). Each of these transcriptional regulators has downstream consequences such as suppression of Interferon regulatory factor 1 (IRF1), another transcriptional regulator, and ERAP1, also known as Type 1 Tumor Necrosis Factor Receptor Shedding Aminopeptidase Regulator. In addition to this, IPA also predicted the downregulation of NUPR1 to inhibit the activation of a number of histone-associated proteins such as H1-5, H2AC4, and H3C12. Further functional studies into the effects of these altered networks could give us an insight into the MALAT1 derived mechanism of NAC resistance in TNBC. Further analysis shows aberrant expression patterns in effectors contributing to angiogenesis. These include factors such as Insulin-like growth factor-binding protein 1 (IGFBP1), the granulin precursor (GRN) and angiogenin, a crucial mediator for new blood vessel formation (Fig. 5e). IPA also revealed suppression of oxidative phosphorylation canonical pathway (Z score = −2, Fig. 5f) in MALAT1-depleted TNBC, further providing novel insight into effects of MALAT1-depletion on canonical pathways in TNBC.

### Alterations in lncRNA transcriptome in MALAT1-KO TNBC models

**Fig. 6:**
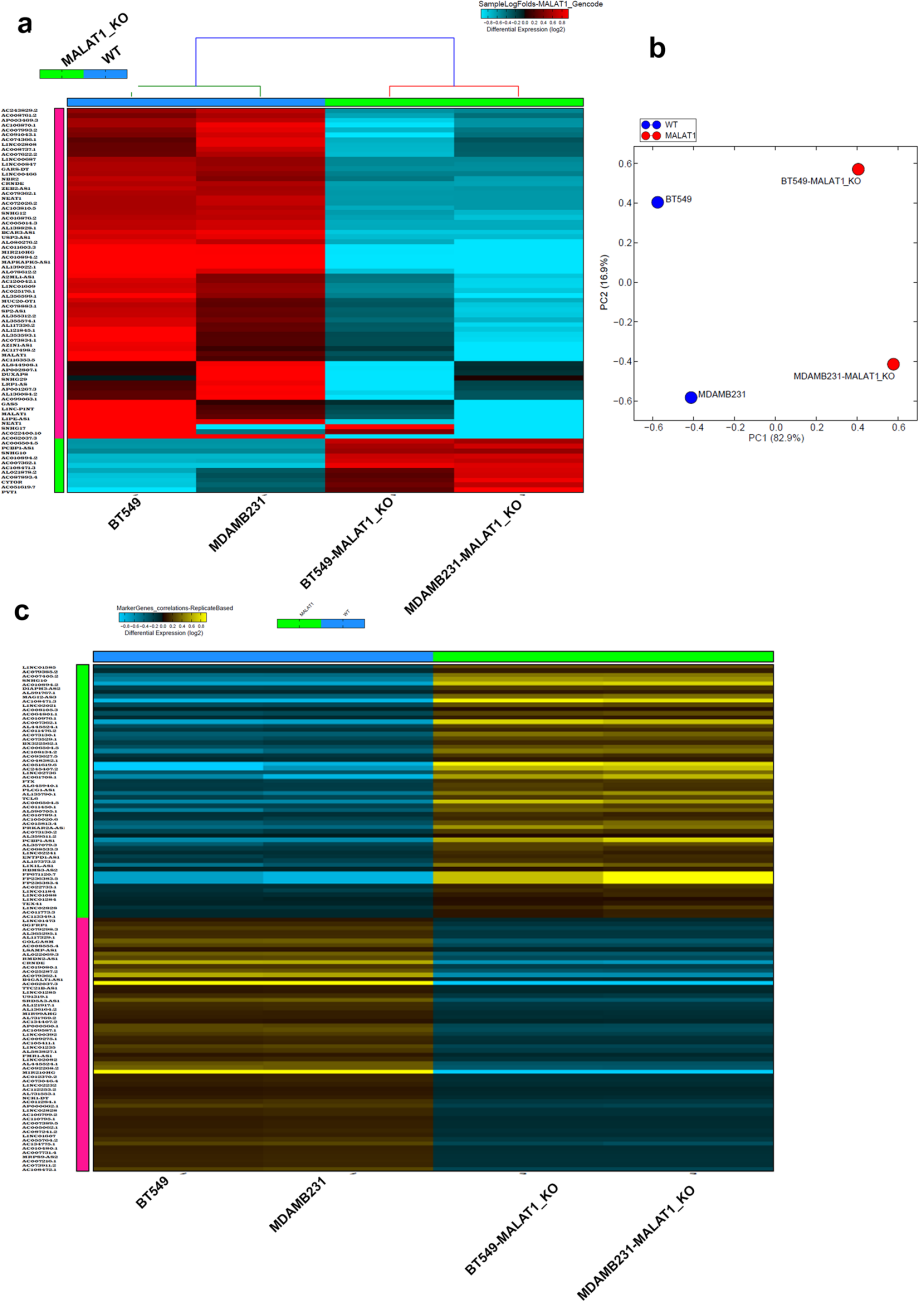
LncRNA transcriptome alterations in MALAT1-KO TNBC models. BT-549-MALAT1-KO, MDA-MB-231-MALAT1-KO, and corresponding parental lines were subjected to whole transcriptome and gencode v33 lncRNA analysis. **a** Hierarchical clustering of BT-549-MALAT1-KO, MDA-MB-231-MALAT1-KO and corresponding parental TNBC models based on lncRNA transcriptome. Each column represents one cell and each row represents lncRNA transcript. The expression level of each transcript (log2) in each sample is depicted according to the color scale. **b** Principal component analysis (PCA) illustrating the segregation of BT-549, BT-549-MALAT1-KO, MDA-MB-231, and MDA-MB-231-MALAT1-KO based on PC1 and PC2. **c** Heatmap depicting the most significant lncRNAs associated with the MALAT-KO vs parental phenotype. Expression is depicted according to the color scale.

Drawing back to our focus on lncRNAs, whole transcriptome data from BT-549-MALAT1-KO, MDA-MB-231-MALAT1-KO, and their parental lines were subjected to alignment and abundance estimate based on the latest gencode v33 lncRNA assembly containing ~48,438 lncRNA transcripts. The expression status of several lncRNAs was found to be affected by the knockout of MALAT1, as shown in the hierarchical clustering in Fig. 6a and Supplementary Table 6. Columns show each cell model type and each row represents a differentially expressed lncRNA transcript (log2). For both BT-549 and MDA-MB-231 MALAT1-KO models, a copious number of lncRNAs were downregulated, while a few transcripts were also upregulated. This is also reflected in the corresponding PCA, which shows clear segregation of each of our cell line models based on PC1 and PC2 (Fig. 6b). The data subsequently were subjected to the marker finder algorithm, which tends to identify the most differential lncRNAs associated with the MALAT1-KO vs wild type phenotype (Fig. 6c). These data highlight lncRNA MALAT1 as a master regulator of a number of other ncRNAs and draws emphasis on its potential importance and relevance in mechanisms involved in NAC resistance in TNBC.

**Fig. 7:**
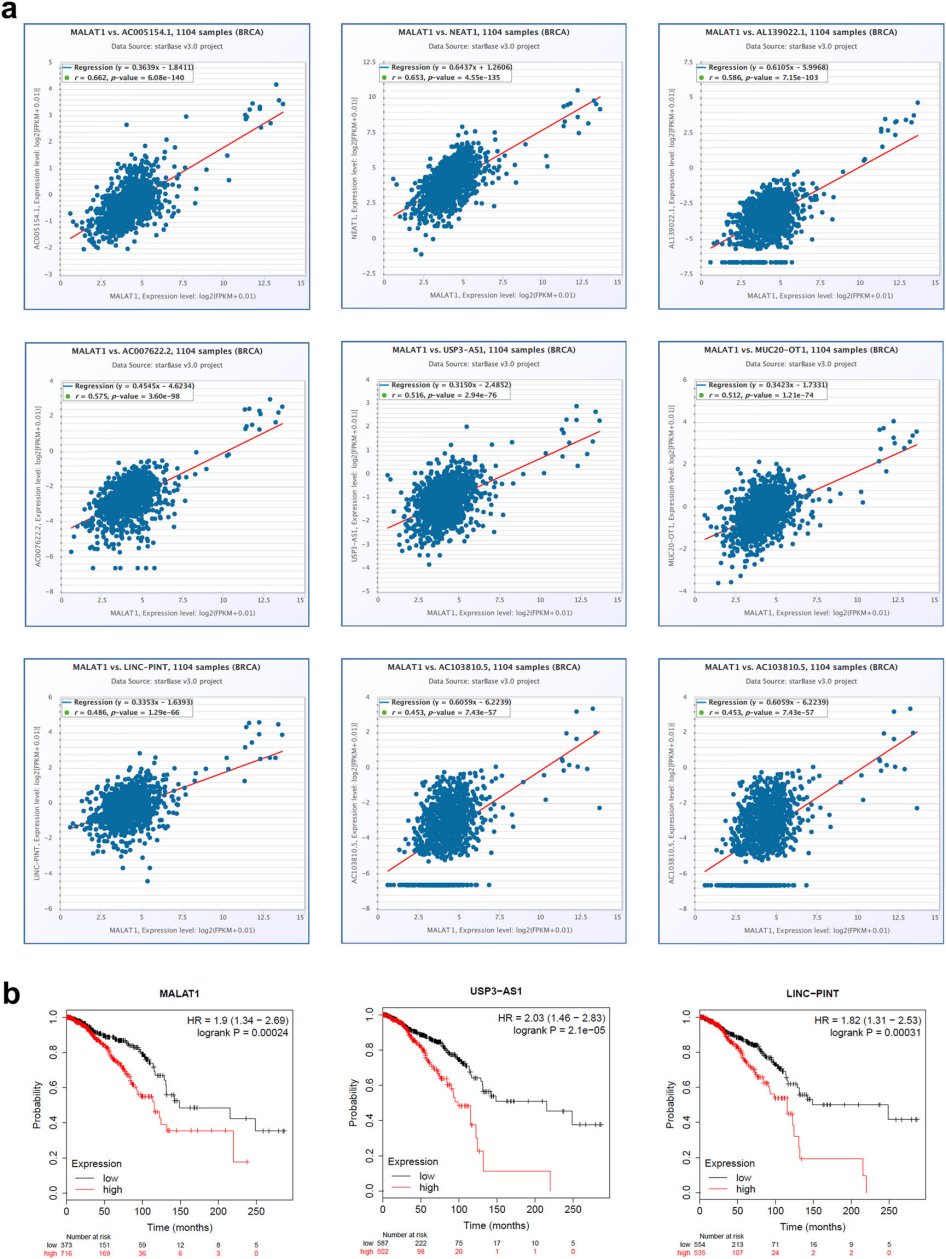
Correlation between MALAT1 expression and the expression of MALAT-1 related lncRNA identified from the current study in independent BC cohort. **a** Correlation between MALAT1 expression and the expression of AC005154.1, NEAT1, AL139022.1, AC007622.2, USP3-AS1, MUC20-OT1, LINC-PINT, and AC103810.5 in 1104 BC patients from the StarBase database. **b** Overall survival (OS) as a function of MALAT1, USP3-AS1, and LINC-PINT expression in breast cancer. The number of patients in each category is indicated on the plot.

To further support our findings from MALAT1-KO whole transcriptome analysis, we subsequently explored the correlation between MALAT1 and the expression of a number of the downregulated lncRNAs, employing a cohort of 1104 breast cancer patients from the StarBase database. Our data showed strong positive correlations between MALAT1 expression and the expression of AC005154.1, NEAT1, AL139022.1, AC007622.2, USP3-AS1, MUC20-OT1, LINC-PINT, and AC103810.5 (Fig. 7a), highlighting a plausible reciprocal relationship between MALAT1 and the identified lncRNAs in TNBC. Interestingly, the expression of MALAT1, USP3-AS1, and LINC-PINT was associated with worse overall survival in breast cancer patients (Fig. 7b).

## Discussion

In the current study, we investigated the lncRNA transcriptional landscape of tumor-derived single-cells in TNBC patients in response to NAC. Kim et al. characterized the two study groups with distinct clonal dynamics as extinction and persistence, those without, and those with persistent residual tumor cells, respectively^16^. The use of single-cell analysis while studying resistance mechanisms is a more constructive way of looking into TNBC due to its widely heterogeneous nature, and shows precise distinctions between the two groups, as well as commonalities in their lncRNA expression profiles.

Several studies have proposed lncRNA signatures that could potentially be used for predictive and prognostic value in response to NAC^17–19^. Wang et al., identified a 36-lncRNA signature in 488 breast cancer patients treated with NAC associated with complete pathological response, using microarray datasets^18^, however the response to TNBC subsets are not specified. A study on 33 paired TNBC and adjacent normal breast tissue found an integrated mRNA-lncRNA signature to predict cancer reoccurrence after paclitaxel based chemotherapy through lncRNA HIF1A-AS2-mediated TNBC proliferation and invasion^19^. Our data show several lncRNAs to be upregulated in persistence cells in comparison to those in the extinction group (CBR3-AS1, RGPD4-AS1, AL356608.1, KCNMA1-AS1, SOX2-OT, LYPLAL1-AS1, AC068831.7, TMEM254-AS1, AC015849.12, and AC015849.3). Countless other studies have associated different lncRNAs to TNBC regulation, progression and even radio-sensitivity^15,20–23^, establishing the oncogenic potential of some lncRNAs, which can serve as targets for therapeutic intervention against TNBC.

To further decipher how aberrant expression of lncRNAs effect NAC resistance, it is useful to understand whether expression patterns suspected in chemo-resistance are pre-existing in the cancer cell population, or if they arise as a consequence of exposure to NAC treatment. Previous work by Kim et al.^16^, suggests a role for both modes of evolution in the establishment of resistance, with the identification of chemo-resistant gene subsets expressed prior to treatment. Our study focused on the yet unexplored role of the lncRNA landscape in single cells of extinction versus persistence groups in response to NAC. In the lncRNA context, a clear shift in transcriptional expression was evident in the pre versus mid NAC cells, however, in concordance with previous findings, multiple cells from the mid NAC group clustered with the pre NAC cells, suggesting they could be the subsets responsible for the observed persistence in TNBC patients and warrant further investigation.

Interestingly, RNAseq data brought to light several upregulated transcripts in the persistence group, all belonging to the MALAT1 locus (ENST00000617791.1, ENST00000610481.1, ENST00000618132.1, ENST00000610851.1, ENST00000534336.1), insinuating a plausible role for MALAT1 in TNBC resistance to NAC. LncRNA MALAT1 has been previously implicated in the progression of several types of cancer including renal cell carcinoma^24^, gall bladder cancer metastasis^25^, and malignant melanoma^26^. Zuo et al. reported the promotion of proliferation and invasion in TNBC through microRNA-129-5p. High MALAT1 expression was linked to poor prognosis and poor overall survival in 43 TNBC patients when analyzing tumor tissue versus non-tumor adjacent tissue. MALAT1 silencing in TNBC cell lines led to induced cell cycle arrest with the inhibition of proliferation, migration, and invasion^27^. In another study by Bamodu et al.^28^, increased expression of MALAT1 correlated with elevated expression of Lysine-specific demethylase 5B protein (KDM5B) through association with hsa-miR-448, promoting an aggressive breast cancer phenotype. MALAT1 has also been shown to function through the miR-1/slug axis, exerting oncogenic activity^29^. Such work supports our findings in the current study, confirming the potential use of MALAT1 expression manipulation as a target for TNBC therapy. In this study, our BT-549 MALAT1-KO model formed relatively fewer colonies when challenged with serial dilutions of Paclitaxel or Doxorubicin compared to the wt, suggesting a plausible role for MALAT1 mediated NAC resistance. The effect of targeting MALAT1 on TNBC chemosensitivity was modest, although reproducible. It is plausible that the modest effect observed is due to the fact that MALAT1 promoter depletion led to ~50% reduction in MALAT1 expression, hence the lack of complete depletion of MALAT1 might minimize its contribution to chemosensitivity.

Whole transcriptome analysis highlighted several transcriptional regulator networks to be suppressed in MALAT-KO cells compared to the wt including NUPR1, STAT1, RELA, and SREBF1. Interestingly, Sterol Regulatory Element Binding Transcription Factor 1 (SREBF1) was shown to interact with MALAT1 to stabilize nuclear proteins, promoting hepatic steatosis and insulin resistance in type 2 diabetes^30^. Li et al.^31^ previously reported that MALAT1 could play a role in inflammatory cytokine release suppression via sponging miR-155, leading to a stunted JAK/STAT pathway in atherosclerosis. Further research into each of these transcriptional regulator networks needs to be conducted to better outline their roles in the onset of TNBC NAC resistance. Differentially expressed genes in 26 TNBC patients receiving NAC show chromatin remodeling as one of the most significantly enriched biological processes according to functional annotations^17^. This is in concordance with our data from ingenuity pathway analysis in MALAT1-KO cells, where several histone-associated proteins are predicted to be inhibited, including H1-5, H2AC4, and H3C12. These downstream effects on the state of chromatin could possibly govern the transcriptional landscape of TNBC subsets, conferring resistance to treatment through MALAT1 expression.

Our data also reveal a strong correlation between the expression of lncRNA MALAT1 and several other lncRNAs expression profiles. We suggest a possible reciprocal relationship between MALAT1 and each of AC005154.1, NEAT1, AL139022.1, AC007622.2, USP3-AS1, MUC20-OT1, LINC-PINT, and AC103810.5. In fact, several studies on NEAT1 lncRNA have shown that upregulated NEAT1 facilitated tumor cell viability, invasion, and migration in breast cancer^32^. In Non-small-cell lung carcinoma, Shikonin mediated NEAT1 suppression resulted in apoptotic cell death of paclitaxel-resistant cell lines^33^. In another study, lncRNA NEAT1 was shown to facilitate the progression of gallbladder cancer via the upregulation of Survivin expression, suggesting its potential use as a biomarker in gallbladder cancer^34^. Multiple other studies show NEAT1 associations with osteoarthritis^35^, lung cancer^36^, and acute myeloid leukemia^37^. USP3-AS1 has also been reported as a potential biomarker for neuroblastoma pathogenesis studies^38^. Marín-Béjar et al.^39^, on the other hand, found that lncRNA LINC-PINT, through a highly conserved region, acted as a tumor suppressor, downregulating factors associated with the capacity for cell migration. Carlevaro-Fita et al.^40^, subsequently found that mutations in LINC-PINT were tumor causing, through CLC datasets. In our study, the expression of MALAT1, USP3-AS1, and LINC-PINT was associated with worse overall survival in breast cancer patients.

In conclusion, we believe that the use of single-cell analysis to look at aberrant expression patterns in TNBC in response to NAC treatment is a more rigorous and unprecedented approach to identifying specific lncRNAs responsible for the onset of NAC resistance, be they pre-existing prior to NAC, or acquired mid-NAC. Through hierarchical clustering, we identified small subsets of cells from prior NAC treatment to cluster with those mid treatments, suggesting the existence of pre-existing resistance cells that arise through cancer genome instability, which we speculate then undergo expansion when challenged with chemotherapeutic intervention. With genome or transcriptome-wide sequencing, it would not be possible to make such observations, further highlighting the benefits of our approach. Aberrantly expressed lncRNA transcripts highlight an important role for MALAT1 lncRNA in TNBC onset and NAC resistance, confirmed through functional studies on MALAT1-KOs, and by delving deeper into the changes in the transcriptional landscape and associated networks arising as a consequence of downregulated MALAT1 expression.

## Materials and methods

### Dataset and read analysis

Single-cell raw RNA sequencing data were retrieved from the sequence read archive (SRA) database under accession no. SRP114962. Data were retrieved using the SRA toolkit version 2.9.2 as previously described^41^. FASTQ files were subsequently pseudo aligned to the ENSEMBL ncRNA assembly and reads were counted using KALLISTO 0.42.1. Abundance data were subsequently subjected to ICGS, UMAP dimensionality reduction, Principal component analysis (PCA), and hierarchical clustering as described before^42–44^. Gene set enrichment and modeling of gene interaction networks were analyzed using Ingenuity Pathways Analysis (IPA) software (Qiagen).

### TNBC cell culture

Human breast cancer cell lines BT-549 and MDA-MB-231 were maintained in RPMI 1640 Medium (ATCC modification); Catalog number A1049101, and DMEM (Dulbecco’s Modified Eagle Medium) respectively. Both are supplemented with D-glucose 4500 mg/l, 2–4 mM L-glutamine, 10% fetal bovine serum and 1x penicillin–streptomycin (Pen-Strep) (all purchased from Gibco-Invitrogen, Waltham, MA, USA). Cell monolayers grew at 37 ^◦^C in humidified conditions of 5% CO_2_.

### Generation of MALAT1 knockout TNBC models using CRISPR/CAS9 technology

#### Synthesis/construction/purification of MALAT1 guide RNA

Guide RNA (gRNA) sequences targeting MALAT1 promoter region were described before^45^. Two guides, guide-1 (GCTGGGGCTCAGTTGCGTAA) and guide-2 sequence (AGGTTTCTAAAACATGACGG) were in vitro synthesized using the EnGen sgRNA Synthesis Kit, S. pyogenes (NEB# E3322S). The in vitro transcribed guides were purified using Monarch RNA cleanup Kit (NEB# T2040L). Manufacturer’s instructions were followed for both the above-mentioned kits. The MALAT1 gRNA were each eluted in 20 μl of nuclease free water and immediately aliquotted into PCR tubes and stored at −80 °C until use. The concentrations of purified gRNA were measured using NanoDrop 2000 (Thermo Fisher Scientific).

### Cas9 RNP transfection using electroporation

The preparation of the ribonucleoprotein (RNP) complex was performed immediately prior to each transfection. Each gRNA was incubated with recombinant Cas9 protein to form RNP complex. In brief, 1000 ng of each MALAT1 gRNA and 1.5 μl of 20 µM Cas-9 Enzyme (EnGen Spy Cas9 NLS (NEB# M0646M) were mixed by pipetting in a PCR tube and allowed to form RNP complex at room temperature for 20 min. During the incubation, 70–80% confluent TNBC (MDA-MB-231 and BT-549) cells were trypsinized followed by washing with PBS to remove any traces of trypsin. The required (2 × 10^5^) TNBC cells for each transfection were aliquotted into sterile 1.5 ml tubes. The cells were pelleted and washed once with 1× PBS and kept on ice until use. The formed RNP complex was pre-mixed with 20 µl of nucleofector solution (P3 Primary Cell 4D-Nucleofector kit- Lonza# V4XP-3024) and then mixed with (2 × 10^5^ cells) TNBC cells by gentle pipetting. The complex mixture (cells+RNP) was immediately transferred into a well of a 16-well Nucleocuvette and electroporated with pre-selected program, MDA-MB-231, using 4D Nucleofector (Lonza, Switzerland). After electroporation, the TNBC cells were recovered in pre-warmed culture media and plated onto two wells of a 6-well plate until the cells were confluent for downstream assays.

### PCR-based genotyping of MALAT1

Genomic DNA was isolated using the RNA/DNA/Protein purification plus kit (Norgen Biotek Corp, Ontario, Canada) as per the manufacturer’s instructions. The concentrations of DNA were measured using NanoDrop 2000 (Thermo Fisher Scientific, DE, USA). We used AmpliTaq™ DNA polymerase kit (Applied Biosystem, Thermo Scientific, USA) for this study. PCR was performed in a 25-μL volume, comprised 100 ng of DNA template, 2.5 μl PCR Buffer (10X), 0.5 μl dNTPs (10 mM), 0.5 μl primer mix (20 μM) and 0.125 μl AmpliTaq Gold DNA polymerase (5U/ μl). Thermal cycling was performed using the following conditions: 1 cycle 95 °C for 10 min; 35 PCR cycles 95 °C for 15 s, 55 °C for 30 s, 72 °C for 1 min; followed by final extension at 72 °C for 10 min. The PCR products were electrophoresed using a 1.5% agarose-gel/TBE buffer system using Bio-Rad. The Gel images were acquired with a CCD-gel imaging system (Bio-Rad, CA, USA).

### Total RNA library preparation and RNA sequencing

Total RNA samples with a RIN higher than 8 were used as input for the library preparation by using TruSeq Stranded Total RNA Library Prep Gold kit (Cat #: 20020598) from Illumina following the manufacturer’s protocol. In brief, 500 ng of total RNA was subjected to rRNA depletion and then to fragmentation. The first-strand cDNA synthesis was performed with random hexamers and SuperScript II Reverse Transcriptase (Cat#: 18064014) from ThermoFisher Scientific. The second cDNA strand synthesis was performed by substitution of dTTP with dUTP. The double-stranded cDNA is then end-repaired and adenylated. Barcoded DNA adapters were ligated to both ends of the double-stranded cDNA and then amplified. The libraries quality was checked on an Agilent 2100 Bioanalyzer system and quantified using Qubit 2.0 fluorometer (Invitrogen). The libraries were pooled, clustered on a cBot platform, and sequenced on an Illumina HiSeq 4000 at a minimum of 50 million paired end reads (2 × 75 bp) per sample.

### Sanger sequencing

MDA-MB-231 and BT-549 TNBC cell lines were sequenced to confirm MALAT1 promoter deletion in our generated knockout models compared to parental models. The sequencing reaction was performed using the BigDye Terminator v3.1 cycle sequencing kit from ThermoFisher according to the manufacturer’s protocol. In brief, in a volume of 10 μl, we added 100–200 ng of the template to 4 μl of BigDye Terminator v3.1 Ready Reaction Mix and 3.2 pmol of primer. The amplification was carried out in a thermal cycler: 96 °C for 1 min, followed by 25 cycles of 96 °C for 10 s, 50 °C for 5 s and 60 °C for 4 min. The amplified DNA was purified using a mixture of SAM/BigDye XTerminatot beads (45 μl of SAM solution and 10 μl of BigDye XTerminatot bead solution), then was subjected to electrophoresis on the 3730xl DNA analyzer.

### Colony-forming unit (CFU) assay and Paclitaxel/Doxorubicin sensitivity of control and MALAT1-KO BT-549 cells

Both control and MALAT1-KO cells (0.02 × 10^6^ cells /well) were treated with different serially diluted concentrations of paclitaxel/PTX or doxorubicin/DOX drug (60–3.7 nM) or for 7 days. On day 7, cells were fixed with 4% PFA for 5 min followed by washing twice in PBS and stained with crystal violet (0.1% in 10% EtOH) for 10 min at room temperature. The images were taken and compared with control without drug treatment. After the wells air dried at room temperature, CFUs were quantified by dissolving crystal violet in 5% SDS and absorbance measured at 590 nm. Data are represented as mean ± S.D. from four technical replicas.

### Statistical analysis

Statistical analyses and graphing were performed using Microsoft excel 2016 and GraphPad Prism 8.0 software (GraphPad, San Diego, CA, USA). Two-tailed *t*-test was used for comparative groups. *P*-values ≤ 0.05 (two-tailed *t*-test) were considered significant. For IPA analyses, a Z score (−2.0 ≤ *Z* ≥ 2.0) was considered significant.

## Supplementary information

Supplementary table 1

Supplementary table 2

Supplementary table 3

Supplementary table 4

Supplementary table 5

Supplementary table 6
